# Exosomal microRNA-205 is involved in proliferation, migration, invasion, and apoptosis of ovarian cancer cells via regulating VEGFA

**DOI:** 10.1186/s12935-019-0990-z

**Published:** 2019-11-07

**Authors:** Lijun Wang, Fei Zhao, Zhongqing Xiao, Liang Yao

**Affiliations:** Department of Oncology, Jiangxi Maternal and Child Health Hospital, No.318 Bayi Avenue, Nanchang, 330000 Jiangxi China

**Keywords:** Exosome, Ovarian cancer, MicroRNA-205, Vascular endothelial growth factor A, Proliferation, Apoptosis, Migration, Invasion

## Abstract

**Background:**

Recently, the impact of microRNAs (miRNAs) and exosome on ovarian cancer has been assessed in many studies. We aim to explore the mechanism of exosomes transferring miR-205 in ovarian cancer, and confirm its diagnostic value in ovarian cancer.

**Methods:**

The expression of miR-205 of ovarian cancer patients and healthy people was detected by RT-qPCR, and the diagnostic value of miR-205 was evaluated. The exosomes derived from SKOV3 cells were identified. Ovarian cancer SKOV3 donor cells and receptor cells were used to measure the proliferation, migration, invasion, apoptosis and cell cycle by a series of experiments. The binding site between miR-205 and vascular endothelial growth factor A (VEGFA) was evaluated by bioinformatics tool and dual-luciferase reporter gene assay.

**Results:**

MiR-205 was up-regulated in ovarian cancer, and up-regulated miR-205 could enhance the risk of ovarian cancer and was one of its risk factors. After SKOV3 cells-derived exosomes were transiently introduced with miR-205 mimics, the cell proliferation, migration and invasion in ovarian cancer were elevated, the apoptosis of ovarian cancer cells was attenuated, and the epithelial–mesenchymal transition (EMT) protein E-cadherin was down-regulated, while Vimentin was elevated. VEGFA was identified to be a target gene of miR-205.

**Conclusion:**

This study suggests that exosomes from donor ovarian cancer cell SKOV3 shuttled miR-205 could participate in the regulation of the proliferation, migration, invasion, apoptosis as well as EMT progression of receptor SKOV3 cells by targeting VEGFA.

## Background

Among all gynecologic malignancies, ovarian cancer is the primary cause of death. Though the therapy of ovarian cancer has been promoted, the current treatment was limited due to the occurrence of chemotherapy-resistant cancer cells [1]. The lack of an obvious, particular clinical presentation in the early stage and the deficiency of screening tool caused that the patients with ovarian cancer could only be diagnosed at more advanced stages. Current treatment could not cure the disease, and the 5-year survival rate of ovarian cancer is 45% [2]. According to the demonstration of previous studies, some of the risk factors had been verified, such as family history of breast cancer [3], circulating vitamin D [4] and central adiposity [5]. In recent years, several microRNAs (miRNAs) have been verified in human diseases, and many extant studies have unraveled that miRNAs were involved in ovarian cancer progression, such as miR-200 [6], miR-15a, miR-16 [7] and miR-506 [8].

MiRNAs are known as small non-coding RNAs and generally deregulated in cancers. As one of the miRNAs, miR-205 is usually silenced in advanced stage of cancers [9]. Additionally, the impacts of miR-205 on human diseases such as advanced pancreatic cancer [10], head and neck squamous cell carcinoma [11] and primary cutaneous T-cell lymphomas [12] have been assessed. Beyond that, Li et al. have found in their study that miR-205 was implicated in the development of ovarian cancer [13]. Wei et al. have found that miR-205 expression increased in ovarian cancer tissues and ovarian cancer cell lines, and overexpressed miR-205 could accelerate the invasion of ovarian cancer cells [14]. Furthermore, Wang et al. have reported that plasma miR-205-5p significantly overexpressed in ovarian cancer patients in comparison with normal controls [15]. In addition, it has been demonstrated by Zhang et al. that the sequence divergent miR-205 was independently overexpressed in mesenchymal-like ovarian cancer cells [16]. These data suggested that miR-205 is highly expressed in ovarian cancer, and the expression of miR-205 was positively related to the proliferation, invasion, and migration of ovarian cancer cells. As for the target relation between miR-205 and vascular endothelial growth factor A (VEGFA), a previous study has mentioned that miR-205 was up-regulated in ovarian cancer cells exposed to VEGF, and miR-205 promoted the invasion and proliferation of ovarian cancer cells [17]. Moreover, exosomes are ceramide-enriched vesicles, with a diameter of 40–100 nm, developed by endosomal membrane inward budding, and were secreted from the fusion of multivesicular endosomes and the plasma membrane. Exosomes could transmit signaling factors as well as miRNAs that mediate intercellular communication [18], and the impact of exosomes on the progression of blood-based ovarian cancer has been discussed [19]. However, the relation among miR-205, exosomes as well as ovarian cancer has not been studied yet, hence, our research was carried out to investigate the capacity of miR-205 and exosomes in ovarian cancer, and we inferred that miR-205 may act as a proto-oncogene in the development of ovarian cancer. Exosomes from donor ovarian cancer cell SKOV3, miR-205 and VEGFA could participate in the progression of ovarian cancer.

## Materials and methods

### Ethics statement

The study was ratified by the Ethics Committee of Jiangxi Maternal and Child Health Hospital and based on the ethical principles for medical research involving human subjects of the *Helsinki Declaration*. Informed written consent was obtained from all the patients.

### Study subjects

A total of 80 patients with ovarian cancer (with the age of 20–72 years old) who have undergone surgical treatment in the department of gynaecology in Jiangxi Maternal and Child Health Hospital from June 2017 to June 2018 were collected as a case group. The specimens of whom were all confirmed by histopathology. The specimens were staged on the basis of the criteria of surgical-pathological stage of Federation International of Gynecology and Obstetrics (FIGO): 28 cases at I stage, 18 cases at II stage, 32 cases at III stage and 2 cases at IV stage; pathological types: 48 cases of serous carcinoma, 20 cases of clear cell carcinoma, 10 cases of mucinous carcinoma and 2 cases of endometrioid carcinoma. A number of 80 healthy females who were at the matching age with patients in the case group were randomly collected as a control group. Patients in the case group have not been treated with radiotherapy, chemotherapy or hormonotherapy before the surgery. The morning fasting venous blood (5 mL) of all subjects were centrifuged and the serum was preserved at − 70 °C for the following experiments.

### Cell culture

Human ovarian cancer cell line SKOV3 was obtained from the cell resource center of Shanghai Institute of Nutrition and Health, Chinese Academy of Sciences (Shanghai, China) and was generally cultured in an incubator at 37 °C and 5% CO_2_, then incubated in RPMI-1640 medium containing 10% fetal bovine serum (FBS, Gibco, Grand Island, NY, USA) with a concentration of 100 U/mL of penicillin and 100 μg/mL of streptomycin. The density of adherent SKOV3 cells was observed under an inverted microscope, and the cells were passaged when the cell confluence reached 90%. Cells in the logarithmic growth phase were harvested for the follow-up experiments.

### Exosome separation and identification

The supernatant was collected after a 48-h cell culture. Based on a Ref. [20], the exosomes were extracted by gradient centrifugation of the supernatant (300 g for 10 min, 1200 g for 20 min, 10,000 g for 30 min) at 4 °C, then the supernatant was centrifuged at 100,000*g* at 4 °C for 1 h, and the sediments were exosomes, which were rinsed by phosphate buffered solution (PBS), then centrifuged at 100,000*g* at 4 °C for 1 h and the sediments were resuspended by PBS and filtered by a 0.22 μm filter to obtain the exosome initial solution, which was preserved at − 80 °C for the following experiments. The morphology and size of exosomes were observed under an electron microscope, and its related protein expression was assessed by Western blot analysis.

### Exosome uptake experiment

The exosomes were marked by PKH67 Fluorescent Cell linker kits (Sigma, St. Louis, MO, US) according to its direction, and the exosomes marked by PKH67 were acquired. A number of (0.5–1) × 10^5^ SKOV3 cells were seeded into 24-well plates and incubated at 37 °C, with 5% CO_2_. The exosomes marked by PKH67 as well as SKOV3 cells were co-cultured without light for 12 h and washed by PBS for three times, 5 min/time, then fixed by paraformaldehyde for 20–30 min, rinsed by PBS for three times, 5 min/time; the nuclei were stained by 2,4-diamino-5-phenylthiazole (DAPI) (Beyotime Biotechnology Co., Ltd., Shanghai, China) for 5 min, rinsed by PBS for three times (5 min/time), and fixed. The distribution of fluorescence was observed by a laser scanning microscope (Nikon Co., Ltd., Tokyo, Japan).

### The role of GW4869 inhibitor in exosome development

Cells in the logarithmic growth phase were seeded onto 24-well plates at 1 × 10^5^ cells/well and incubated. The 24-well plates seeded with SKOV3 cells were took out 24 h in advance with medium discarded, then added with 14.5 μL GW4869 storage solution, 1.5 μL dimethyl sulfoxide (DMSO) solution and RPMI-1640 complete culture solution containing 10% FBS, making the concentration of GW4869 in each well reached 10 μM, and cells supplemented with 0 μM GW4869 were taken as the Mock group. After 48-h incubation, the total RNA was extracted from the treated cells, and miR-205 expression in supernatant and cells was evaluated using reverse transcription quantitative polymerase chain reaction (RT-qPCR).

### Cell grouping and transfection

Ovarian cancer cell line SKOV3 in the logarithmic growth phase was adopted and the cells were separated into three groups: the blank group: cells without transfection; the mimics negative control (NC) group: cells transfected with miR-205 mimics NC or Cy3-mimics NC; the miR-205 mimics group: cells transfected with miR-205 mimics or Cy3-miR-205 mimics. Cy3-miR-205 mimics, Cy3-mimics NC, miR-205 mimics and mimics NC were all obtained from Guangzhou RiboBio Co., Ltd. (Guangdong, China). Cy3-miR-205 mimics, Cy3-mimics NC or miR-205 mimics and mimics NC were transfected by Lipofectamine™ RNAiMAX (Invitrogen, Carlsbad, CA, USA) on the basis of the kit instruction.

### Establishment of cell co-culture models

SKOV3 cells that have transfected with Cy3-miR-205 mimics and Cy3-mimics NC for 36 h (the current SKOV3 cells were donor cells) were collected and seeded at 1 × 10^5^ cells/well in the apical chamber of the transwell plate (the membrane pore size was 0.4 μm), the complete medium was made up to 300 μL. The basolateral chamber was seeded with generally cultured SKOV3 cells (the current SKOV3 cells were receptor cells) 1 day in advance and at 1 × 10^5^ cells/well, three wells were set in each group. After 24-h culture of the cells in both apical chamber and basolateral chamber, the entry of Cy3-miR-205 mimics and Cy3-mimics NC into receptor cells was observed by a FSX100 intelligent biological navigator (Olympus, Tokyo, Japan); the receptor cells were harvested and the total RNA was extracted, then miR-205 expression in the receptor cells was measured by RT-qPCR. The exo-blank group: donor SKOV3 cells without any transfection were co-cultured with receptor SKOV3 cells; the exo-mimics NC group: donor SKOV3 cells were transfected with Cy3-mimics NC and co-cultured with receptor SKOV3 cells; the exo-miR-205 mimics group: donor SKOV3 cells were transfected with Cy3-miR-205 mimics and co-cultured with receptor SKOV3 cells.

### 5-Ethynyl-2′-deoxyuridine (EdU) assay

The donor SKOV3 cells and receptor SKOV3 cells were co-cultured for 24 h and the DNA replication ability of receptor SKOV3 cells in each group was measured by Cell-light EdU kit (Ribo Bio Co., Ltd., Guangzhou, China). The steps are as follows: the cells of each group were incubated by 100 μM EdU solution for 2 h and fixed by 4% paraformaldehyde for 20 min, then 2% glycine was added for 15-min incubation, the cells were washed by PBS two times and added with 150 μL penetrant for permeabilization, and the following steps were conducted under the guide of the direction of EdU kit. Five fields of view were randomly photographed by a fluorescence microscope (FSX100, Olympus, Tokyo, Japan), the blue fluorescence represented for all the cells, and the red fluorescence represented for the replicating cells that have been penetrated by EdU. The proliferation rate of receptor SKOV3 cells was counted as the number of cells with blue fluorescence/the number of cells with red fluorescence × 100%.

### Flow cytometry

#### Cell cycle detection

The receptor SKOV3 cells that have been co-cultured for 24 h were collected and resuspended by PBS, the cell concentration was adjusted to 1 × 10^6^ cells/mL and the cells were made into single cell suspension, centrifuged at 2000 rpm for 5 min to remove the supernatant. Each group was added with 500 μL 70% cold ethanol and fixed at 4 °C for 2 h. With the stationary liquid discarded, the cells were added with 1 mL PBS and centrifuged at 2000 rpm for 3 min; the cells were added with 100 μL RNase A (100 μg/mL) for water bath at 37 °C for 30 min, stained with 400 μL propidium iodide (PI) (50 μg/mL) at 4 °C for 30 min without light exposure, then detected by the machine, and the red fluorescence at 488 nm excitation wavelength was recorded.

Apoptosis detection: after 24-h co-culture, the receptor SKOV3 cells in each well were added with 1 mL serum-free RPMI-1640 medium, then 24-h starvation was followed by trypsinization of trypsin without ethylene diamine tetraacetic acid (EDTA). Subsequently, the cells were collected after 5-min centrifugation at 2000 rpm, washed twice by pre-cold PBS and centrifuged. After resuspended in 500 μL binding buffer, cells in each well were added with 10 μL AnnexinV-FITC (20 μg/mL) and 5 μL PI (20 μg/mL) for fluorescence labeling, then mixed up and incubated for 15 min. The cell apoptosis was evaluated by flow cytometry in 1 h. Judgment of the results: AnnexinV was taken as abscissa axis and PI wad taken as vertical axis; cells in the left upper quadrant were mechanical damaged cells; cells in the right upper quadrant were advanced apoptotic or necrotic cells; cells in the left lower quadrant were negative normal cells; cells in the right lower quadrant were viable apoptotic cells.

### Cell migration experiment

The receptor SKOV3 cells that have been co-cultured for 24 h were collected and resuspended by serum-free RPMI-1640 medium, then seeded into the apical chamber of transwell at a density of 5 × 10^3^ cells/well, the medium was made up to 150 μL and the basolateral chamber was added with 600 μL RPMI-1640 complete medium without antibiotic. After 24-h culture at 37 °C and 5% CO_2_, the cells were fixed by 95% ethanol for 10 min, stained by crystal violet dye for 20 min, then observed by an intelligent biological navigator (Olympus, Tokyo, Japan), five random fields of view were adopted and the number of transmembrane cells were counted.

### Cell invasion experiment

Matrigel was melted at 4 °C overnight and diluted by pre-cold serum-free RPMI1640 medium (at a ratio of 8: 1); the medium (50 μL) was paved on the transwell polycarbonate membrane with a pore diameter of 8 μm, making all the wells were covered by Matrigel at 37 °C for 2 h. The receptor SKOV3 cells that have been co-cultured for 24 h were collected and resuspended by serum-free RPMI-1640 medium, then seeded into the apical chamber of transwell at 1 × 10^5^ cells/well, the medium was made up to 150 μL. The basolateral chamber was added with 600 μL RPMI-1640 complete medium containing 50% FBS. After 24-h culture at 37 °C and 5% CO_2_, the cells were fixed using 4% paraformaldehyde for 15 min, stained by crystal violet dye for 10 min, five random fields of view were photographed and the number of transmembrane cells was recorded.

### RT-qPCR

The total RNA was extracted by SunShine Bio™ kit (SunShine Bio Co., Ltd., Nanjing, China). The total RNAs of the cells and the exosomes were extracted by Trizol (Invitrogen, Carlsbad, CA, USA). The concentration and purity of RNA (the ratio of A260/A280) were evaluated on NANODROP 2000C (Thermo Fisher Scientific Co., Ltd., MA, USA), the ratio of RNA purity at 1.8–2.0 was eligible. The primers of miR-205 and U6 as well as PCR primers were all purchased from Guangzhou RiboBio Co., Ltd. (Guangdong, China), the primer sequence was not provided for business factors. The reverse transcription of VEGFA and β-actin was conducted by MMLV kit (Invitrogen, Carlsbad, CA, USA), PCR primers were synthetized by the Shanghai branch of Invitrogen (Shanghai, China). VEGFA primers are as follows: Forward: 5′-ACGGATCCATGGCGGTCCCACGTC-3′, Reverse: 5′-TTGAATTCTTACCGCCTCGGCTTGTCAC-3′. β-actin primer are as follows: Forward: 5′-ATCCGCAAAGACCTGT-3′, Reverse: 5′-GGGTGTAACGCAACTAAG-3′. The data were analyzed using 2^−ΔΔCt^ method.

### Western blot analysis

Proteins of the cells and the exosomes were extracted and the concentration was measured according to the instruction of bicinchoninic acid kit (Boster Biological Technology Co., Ltd., Wuhan, Hubei, China), the extracted proteins were added with buffer and boiled at 95 °C for 10 min (30 μg/well). Then electrophoresis separation was conducted by 10% polyacrylamide gel (Boster Biological Technology Co., Ltd., Wuhan, Hubei, China), the proteins were transferred onto polyvinylidene fluoride (PVDF) membrane and sealed with 5% bovine serum albumin (BSA) for 1 h, added with primary antibodies CD9 (1: 2000), CD63 (1: 1000), TSG101 (1: 1000), VEGFA (1: 10,000), E-cadherin (1: 1000), Vimentin (1: 1000) and β-actin (1: 5000, all from Abcam, Cambridge, UK), PCNA (1: 1000), cyclin D1 (1: 1000, both from Santa Cruz Biotechnology, Santa Cruz, CA, USA), p-AKT (1: 1000), AKT (1: 1000), p-mTOR (1: 1000), mTOR (1: 1000), Bax (1: 1000), Bcl-2 (1: 1000, all from Cell Signaling Technology, Beverly, MA, USA), MMP-2 (1: 1000), and MMP-9 (1: 1000, both from Proteintech, Chicago, Illinois, USA), and incubated at 4 °C overnight, and rinsed by tris buffer solution with tween (TBST), 3 times/5 min. The relative secondary antibodies (Southern Biotech Co., Ltd., AL, USA) were incubated for 1 h and developed by chemiluminescence reagent. β-actin was taken as an internal reference. The proteins were developed by Gel Doc EZ imager (Bio-rad, CA, USA), and Image J software was used for the grey value analysis of the target band.

### Dual-luciferase reporter gene assay

The online prediction software http://www.targetscan.org was adopted to verify the target sites of VEGFA and miR-205, the sequence was designed and synthetized by GenScript Co., Ltd. (Nanjing, China). The obtained target products as well as pMIR-REPORT™ Luciferase carrier vector were conducted with double digestion, and digestion products of restriction enzyme Hind III and Spe I were recycled, then connected by T4 DNA ligase. The Escherichia coli DH5α and competent cells were transformed, and the plasmid was extracted, then the right recombinant plasmid was acquired. SKOV3 cells were seeded onto 12-well plates at 1 × 10^5^ cells/well, which were co-transfected with recombinant plasmid and miR-205 mimics for 48 h with medium discarded, each well was added with 100 μL cell lysate for 30 min, then 20 μL cell lysate was added with 100 μL LARII and the fluorescence value (A) was measured. The cell lysate was added with 100 μL Stop & Glo reagent and the fluorescence value (B) was detected; fluorescence value (A) was taken as an internal reference, and the luciferase activity value C = B/A.

### Statistical analysis

All data analyses were conducted using SPSS 21.0 software (SPSS, Inc, Chicago, IL, USA). All data was subjected to normal distribution and homogeneity of variance test. The measurement data conforming to the normal distribution were performed as mean ± standard deviation. The unpaired *t*-test was performed for comparisons between two groups and one-way analysis of variance (ANOVA) was used for comparisons among multiple groups, the least significant difference method (LSD) test was use for pairwise comparisons. *P* value < 0.05 was indicative of statistically significant difference.

## Results

### MiR-205 was highly expressed in ovarian cancer

**Fig. 1:**
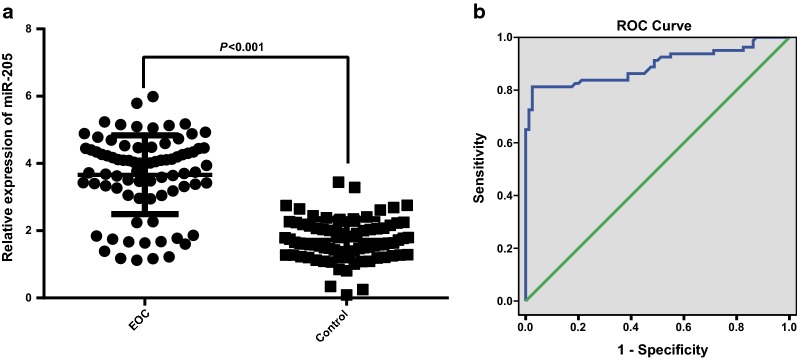
MiR-205 was highly expressed in ovarian cancer. **a** miR-205 expression in the serum of the case group (N = 80) and the control group (N = 80) was detected by RT-qPCR; **b** the diagnostic efficiency of miR-205 expression to ovarian cancer was evaluated by ROC curve; data were analyzed using *t* test

The expression of miR-205 in peripheral blood serum of the two groups was evaluated by RT-qPCR, the results indicated that the miR-205 expression in the case group was noticeably advanced in comparison to the control group (*P* < 0.05, Fig. 1a). To explore the relation between miR-205 expression and the risk of ovarian cancer, miR-205 expression in the case group as well as the control group was analyzed by univariate regression analysis with miR-205 expression as a covariant, the outcomes indicated that the up-regulated miR-205 could increase the risk of ovarian cancer, which was a risk factor, and the odds ratio (OR) was 119.80 (*P* < 0.001), indicating that miR-205 may perform as a tumor promoter in the progression of ovarian cancer.

The diagnosis efficiency of miR-205 expression to ovarian cancer patients was further detected by receiver operating characteristic (ROC) curve, and the outcomes suggested that the area under curve (AUC), sensitivity and specificity were 0.894 (95% confidence interval (CI) was 0.840–0.948), 80.0% and 97.5%, respectively (Fig. 1b), suggesting that there was a good prediction efficiency of miR-205 expression to the tumorigenesis of ovarian cancer.

The relation between miR-205 expression and the clinicopathologic features of ovarian cancer was analyzed, the outcomes indicated that miR-205 expression has nothing to do with age, menopause status and pathological types (all *P* > 0.05). With the development of clinical stages of ovarian cancer, miR-205 expression was gradually enhanced, and miR-205 expression in ovarian cancer patients in III and IV stage was higher than that in I and II stage (both *P* < 0.05). In addition, miR-205 was related to lymphatic metastasis, and miR-205 expression of patients with lymphatic metastasis was evidently higher than those without lymphatic metastasis (*P* < 0.05, Table 1).

**Table 1:** miR-205 expression and clinicopathologic features of patients with ovarian cancer

Clinicopathologic parameter	Case	miR-205 relative expression	*P*
Age (years old)			0.649
> 60	38	3.60 ± 1.28	
≤ 60	42	3.72 ± 1.07	
Menopause			0.110
Yes	44	3.48 ± 1.31	
No	36	3.90 ± 0.93	
Clinical stage			0.011
I + II	46	3.38 ± 1.36	
III + IV	34	4.05 ± 0.71	
Pathological type			0.126
Serosity	48	3.47 ± 1.40	
Mucosity	10	3.62 ± 0.72	
Clear cell	20	4.11 ± 0.52	
Lymphatic metastasis			0.036
No	58	3.50 ± 1.30	
Yes	22	4.11 ± 0.52	

### Exosomes could be assimilated and internalized by ovarian cancer cells

**Fig. 2:**
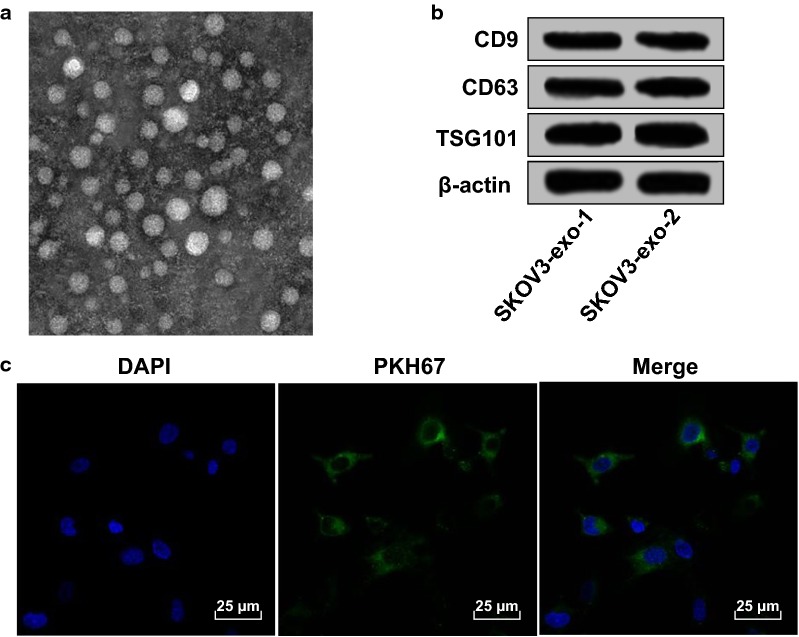
Exosomes could be assimilated and internalized by ovarian cancer cells. **a** Exosomes from SKOV3 cell line observed under a transmission electron microscope; **b** exosome marker proteins CD9, CD63 and TSG101 were detected by Western blot analysis, SKOV3 indicated that the exosomes were derived from SKOV3 cells; **c** uptake of exosomes from SKOV3 observed under a confocal microscopy: PKH67 (green fluorescence) marked the exosomes, DAPI (blue fluorescence) stained the nucleus, the co-localization of fluorescence was observed by Merge

We could find from the centrifugation that the diameter of the micro vesicles that extracted from supernatant of SKOV3 cells was 30–100 nm (Fig. 2a) and the vesicles were oval, which was in consistent with the diameter and feature of exosomes reported by literatures. The expression of specific markers of exosomes (CD9, CD63 and TSG101) was measured by Western blot analysis (Fig. 2b), the outcomes implied that there existed expression of CD9, CD63 and TSG101 in the collected exosomes, suggesting that the collected vesicles were exosomes.

To verify whether the extracted exosomes could be assimilated and internalized by ovarian cancer cells, the exosomes from SKOV3 cells were marked by PKH67 dye, and SKOV3 cells were conducted with stained exosomes for 12 h. The nuclei were stained by DAPI, the distribution and strength of fluorescence were observed under a laser confocal microscope, the results were shown in Fig. 2c. The exosomes marked by PKH67 and in green fluorescence were situated in the cytoplasm of SKOV3 cells, and distributed around the nuclei; green fluorescence could be observed in almost all the cells.

### GW4869 inhibited the generation of exosomes and exosomes mediated the transmission of miR-205 in SKOV3 cells

**Fig. 3:**
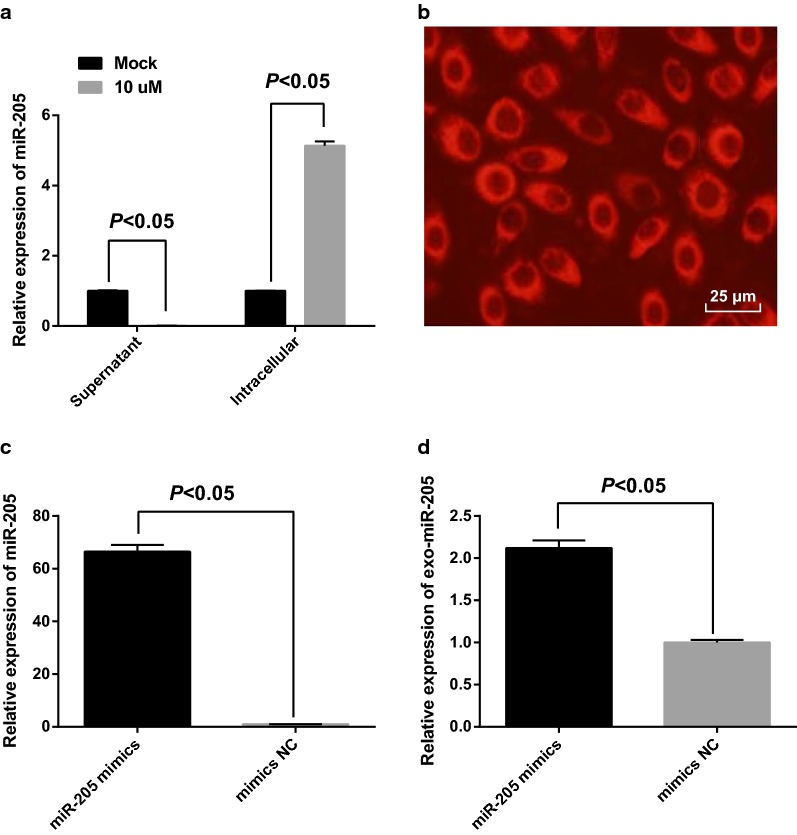
GW4869 inhibited the generation of exosomes and exosomes mediated the transmission of miR-205 in SKOV3 cells. **a** The effect of GW4869 inhibitor on the generation of exosomes; **b** the entry of miR-205 mimics marked by Cy3 into receptor cells; **c** the transfection efficiency of miR-205 mimics; **d** miR-205 expression in the receptor cells

According to the results of RT-qPCR, miR-205 expression in cells as well as supernatant was observably changed after GW4869 was appended into the medium for 48 h. MiR-205 in supernatant that supplemented with 10 μM GW4869 was declined, which was compared with that of supplemented with 0 μM GW4869 (the Mock group) (*P* < 0.05), suggesting that GW4869 could suppress the generation of exosomal miR-205. The decrease of exosomes secreted by cells resulted in an increased miR-205 expression of the 10 μM GW4869 group in contrast to the Mock group (*P* < 0.05, Fig. 3a).

The role of exosomes in receptor cells was analyzed by Transwell assay. The donor SKOV3 cells were in apical chamber and the receptor cells were in the basolateral chamber. After 24-h co-culture of receptor SKOV3 cells and donor SKOV3 cells that have been transfected with Cy3-miR-205 mimics, red fluorescence could be found in most receptor SKOV3 cells (Fig. 3b). The results of RT-qPCR revealed that after 48-h transfection, miR-205 expression in SKOV3 cells of the miR-205 mimics group was 66.48 times higher over the mimics NC group (Fig. 3c), unraveling that miR-205 mimics was of high transfection efficiency. After 24-h co-culture, miR-205 expression of the receptor SKOV3 cells was 2.12 times higher over the mimics NC group (Fig. 3d), indicating that miR-205 from donor SKOV3 cells could transmit into receptor SKOV3 cells via exosomes.

### Overexpressed miR-205 mediated by exosomes promoted proliferation of ovarian cancer cells

**Fig. 4:**
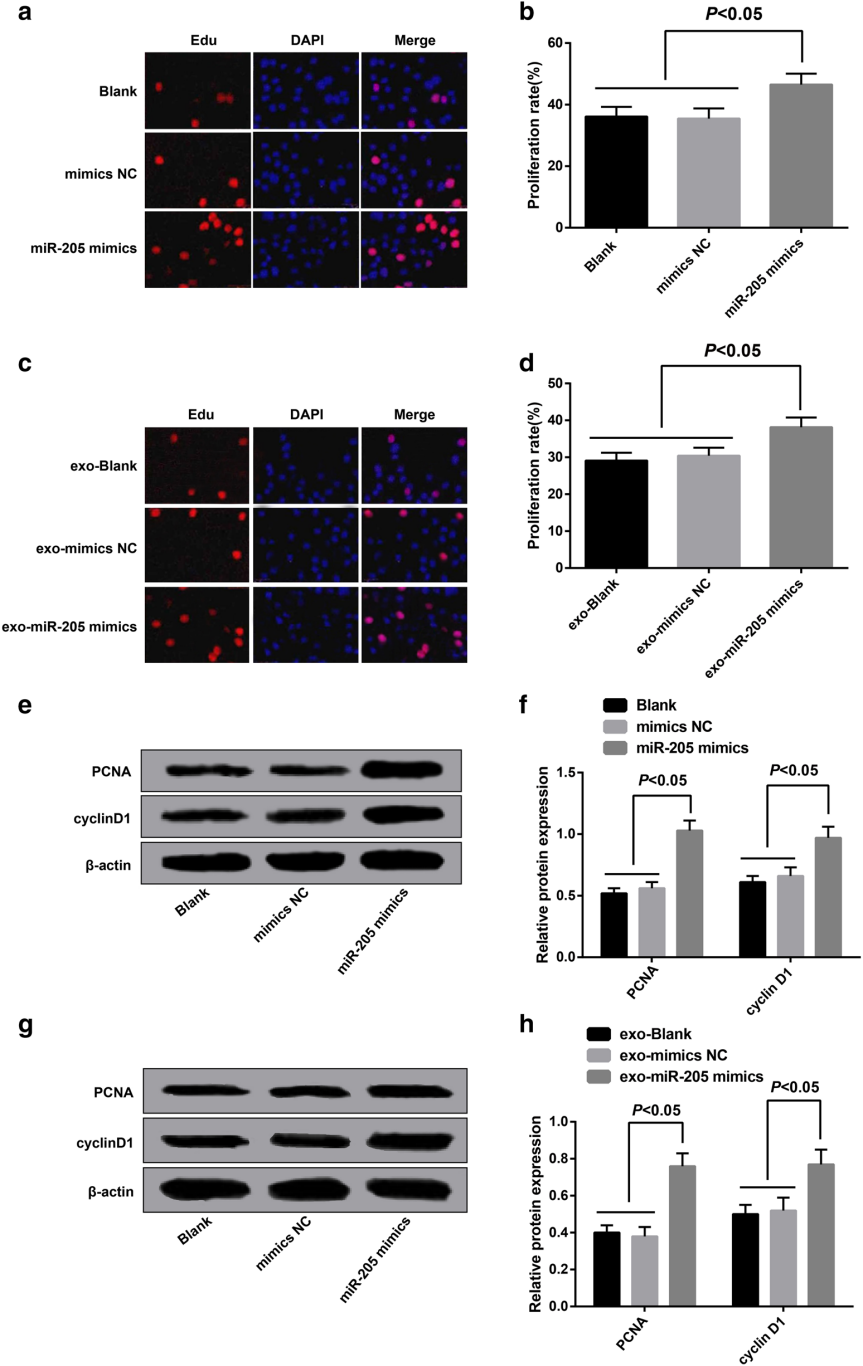
Overexpressed miR-205 mediated by exosomes promoted proliferation of ovarian cancer cells. **a** EdU staining results of SKOV3 cells transfected with miR-205 mimics; **b** the statistical results of proliferation rate of SKOV3 cells transfected with miR-205 mimics in each group; **c** EdU staining results of receptor SKOV3 cells in each group; **d** the statistical results of proliferation rate of receptor SKOV3 cells in each group;**e** protein bands of PCNA and cyclin D1 in SKOV3 cells transfected with miR-205 mimics; **f** the statistical results of protein expression of PCNA and cyclin D1 in SKOV3 cells transfected with miR-205 mimics of each group; **g** protein bands of PCNA and cyclin D1 expression of receptor SKOV3 cells in each group; **h** the statistical results of protein expression of PCNA and cyclin D1 in receptor SKOV3 cells of each group

The cell proliferation of each group was evaluated by EdU assay, the outcomes (Fig. 4a, b) indicated no significant difference could be found in the proliferation rate between the blank group and the mimics NC group (*P *> 0.05); relative to the blank group, the proliferation rate in the miR-205 mimics was advanced (*P *< 0.05), suggesting that overexpressed miR-205 promoted proliferation of ovarian cancer cells.

The receptor cell proliferation of each group was measured by EdU assay, the outcomes (Fig. 4c, d) demonstrated that there was no evident difference in the proliferation rate between the exo-blank group and the exo-mimics NC (*P *> 0.05); in contrast to the exo-blank group, the proliferation rate of SKOV3 cells in the exo-miR-205 mimics group was elevated (*P *< 0.05). These results indicated that exosomes shuttled miR-205 could promote the proliferation of receptor SKOV3 cells.

The expression of proliferation-related proteins (PCNA, cyclin D1) was measured by Western blot analysis, the results suggested that no considerable difference could be observed in the expression of proliferation-related proteins between the blank group and the mimics NC group (*P *> 0.05); in comparison to the blank group, the expression of proliferation-related proteins of the miR-205 mimics was increased (*P *< 0.05, Fig. 4e, f); there was no considerable difference in the expression of proliferation-related proteins between the exo-blank group and the exo-mimics NC group (*P *> 0.05); relative to the exo-blank group, the expression of proliferation-related proteins of the exo-miR-205 mimics was enhanced (*P* < 0.05, Fig. 4g, h).

### Overexpressed miR-205 mediated by exosomes suppressed apoptosis of ovarian cancer cells

**Fig. 5:**
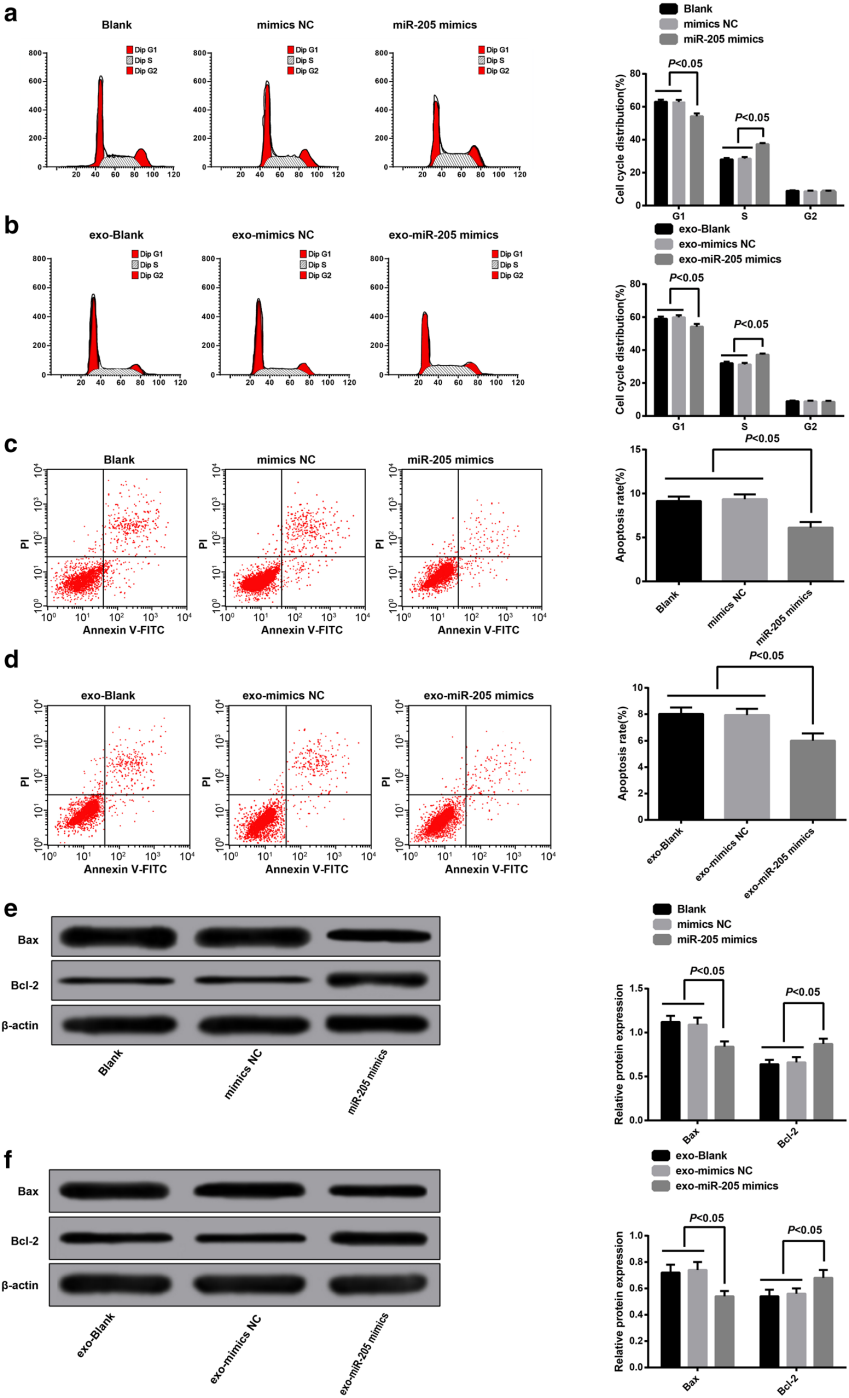
Overexpressed miR-205 mediated by exosomes suppressed apoptosis of ovarian cancer cells. **a** cell cycle detection in each group after SKOV3 cells was transfected with miR-205 mimic; **b** cell cycle detection of receptor SKOV3 cells in each group; **c** apoptosis detection in each group after SKOV3 cells was transfected with miR-205; **d** apoptosis detection of receptor SKOV3 cells in each group; **e** the expression of Bax and Bcl-2 in each group after SKOV3 cells was transfected with miR-205; **f** the expression of Bax and Bcl-2 of receptor SKOV3 cells in each group

The cell cycle distribution of each group was evaluated by flow cytometry, the outcomes revealed that (Fig. 5a) there was no evident difference in cell cycle distribution among the blank group and the mimics NC group (*P *> 0.05), in contrast to the blank group, cells in the G1 stage of the miR-205 mimics group were reduced, while cells in the S stage were relatively increased (*P* < 0.05). We found from these results that overexpressed miR-205 was able to arrest more cells in the S stage of the ovarian cancer and the promote DNA replication activity, contributing to the promotive role of overexpressed miR-205 in ovarian cancer cell proliferation.

The cell cycle distribution of receptor cells in each group was measured by flow cytometry, the results illustrated that (Fig. 5b) no obvious difference could be observed in cell cycle distribution between the exo-blank group and the exo-mimics NC group (*P *> 0.05), contrasted to the exo-blank group, cells in the G1 stage were declined, and cells in the S stage were increased in the exo-miR-205 mimics group (*P* < 0.05). There was no difference among the cells in G2 stage of each group (*P *> 0.05). Above results proved that after donor SKOV3 cells transfected with miR-205 overexpression, exosomes shuttled miR-205 could induce receptor SKOV3 cells arrest at S stage, and also could elevate the DNA reduplication activity, thereby enhancing the ovarian cancer cell proliferation.

The apoptotic outcomes of SKOV3 cells transfected with miR-205 mimics revealed that (Fig. 5c) there was no difference in apoptosis rate between the blank group and the mimics NC group (*P *> 0.05), relative to the blank group, the apoptosis rate of the miR-205 mimics group was decreased (*P* < 0.05). The results unearthed that overexpressed miR-205 could inhibit apoptosis of ovarian cancer cells.

The apoptotic outcomes of receptor cells in each group suggested that (Fig. 5d) no noticeable difference could be observed in cell apoptosis between the exo-blank group and the exo-mimics NC group (*P *> 0.05), while the apoptosis rate of the exo-miR-205 mimics group was reduced, which was contrasted to the exo-blank group (*P* < 0.05). These outcomes confirmed that exosomes shuttled miR-205 could repress apoptosis of receptor SKOV3 cells.

The expression of apoptosis-related proteins (Bax, Bcl-2) was valuated using Western blot analysis. The outcomes illustrated that no significant difference could be observed in the expression of Bax and Bcl-2 between the blank group and the mimics NC group (*P *> 0.05); in comparison to the blank group, the expression of Bax in the miR-205 mimics group was declined, while the expression of Bcl-2 was heightened (*P *< 0.05, Fig. 5e). No obvious difference was performed in the expression of Bax and Bcl-2 between the exo-blank group and the exo-mimics NC group (*P *> 0.05); contrasted to the exo-blank group, the expression of Bax in the exo-miR-205 mimics group was reduced, while the expression of Bcl-2 was enhanced (*P *< 0.05, Fig. 5f).

### Overexpressed miR-205 mediated by exosomes accelerated migration and invasion of ovarian cancer cells

**Fig. 6:**
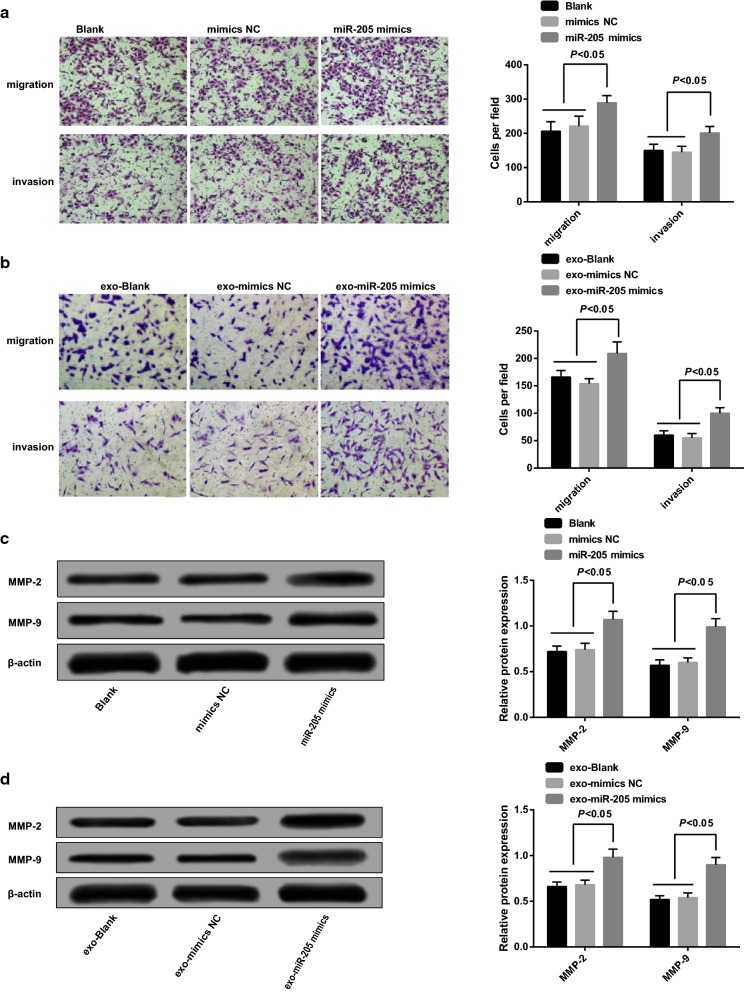
Overexpressed miR-205 mediated by exosomes accelerated migration and invasion of ovarian cancer cells. **a** Migration and invasion detection in each group after SKOV3 cells was transfected with miR-205 mimic; **b** migration and invasion detection of receptor SKOV3 cells in each group; **c** detection of invasion-related proteins (MMP-2, MMP-9) after SKOV3 cells was transfected with miR-205 mimic; **d** detection of invasion-related proteins (MMP-2, MMP-9) of receptor SKOV3 cells in each group

The cell migration of each group was evaluated by Transwell assay, the outcomes of SKOV3 cells introduced to miR-205 mimics revealed that (Fig. 6a) no apparent difference in cell migration could be found between the blank group and the mimics NC group (*P *> 0.05); in contrast to the blank group, the migrated cells in the miR-205 mimics group were increased (*P *< 0.05). The results unraveled that overexpressed miR-205 could promote migration of ovarian cancer cells.

The cell migration of receptor cells in each group was measured by Transwell assay, the results illustrated that (Fig. 6b) no obvious difference could be observed in migrated cells between the exo-blank group and the exo-mimics NC group (*P *> 0.05); in comparison to the exo-blank group, the migrated cells in the exo-miR-205 mimics was enhanced (*P *< 0.05). These outcomes implied that exosomes shuttled miR-205 could promote migration of receptor SKOV3 cells.

The outcomes of cell invasion of SKOV3 cells transfected with miR-205 mimics implied that (Fig. 6a) no evident difference could be observed in cell invasion between the blank group and the mimics NC group (*P *> 0.05); relative to the blank group, the invasive cells in the miR-205 mimics group were increased (*P *< 0.05). The results proved that overexpressed miR-205 could accelerate invasion of ovarian cancer cells.

The cell invasion of each group was evaluated by Transwell assay, the outcomes revealed that (Fig. 6b) no apparent difference in the number of invasive cells could be found between the exo-blank group and the exo-mimics NC group (*P *> 0.05); contrasted to the exo-blank group, the invasive cells in the exo-miR-205 mimics group were increased (*P *< 0.05). The results unraveled that exosomes shuttled miR-205 could promote invasion of receptor SKOV3 cells.

The expression of invasion-related proteins (MMP-2, MMP-9) was measured by Western blot analysis. The results showed that no significant difference was performed in MMP-2 and MMP-9 expression between the blank group and the mimics NC group (*P *> 0.05); in contrast to the blank group, MMP-2 and MMP-9 expression in the miR-205 mimics group was heightened (*P *< 0.05, Fig. 6c). No obvious difference could be observed in MMP-2 and MMP-9 expression between the exo-blank group and the exo-mimics NC group (*P *> 0.05); compared with the exo-blank group, MMP-2 and MMP-9 expression in the exo-miR-205 mimics group was elevated (*P *< 0.05, Fig. 6d).

### Overexpressed miR-205 mediated by exosomes regulated epithelial–mesenchymal transition (EMT) of ovarian cancer cells

**Fig. 7:**
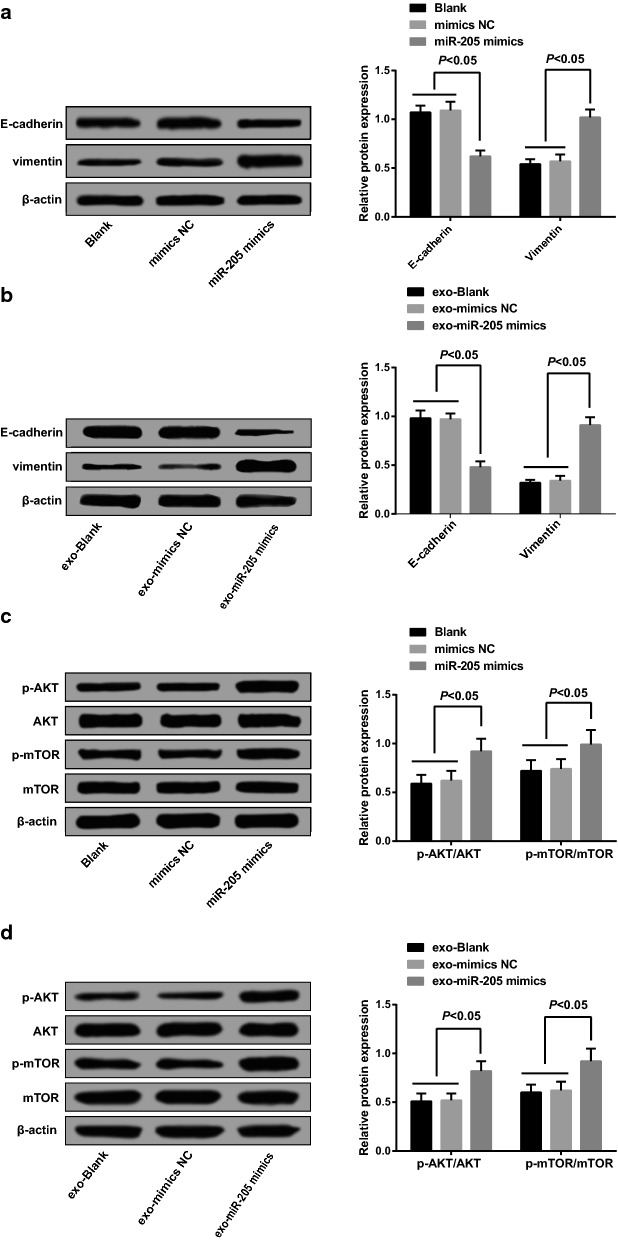
Overexpressed miR-205 mediated by exosomes regulated epithelial–mesenchymal transition of ovarian cancer cells. **a** Detection of EMT proteins E-cadherin and Vimentin in each group after SKOV3 cells was transfected with miR-205 mimic; **b** detection of EMT proteins E-cadherin and Vimentin of receptor SKOV3 cells in each group; **c** expression of p-AKT/AKT and p-mTOR/mTOR in SKOV3 cells of each group after SKOV3 cells were transfected with miR-205 mimics; **d** expression of p-AKT/AKT and p-mTOR/mTOR in receptor SKOV3 cells of each group

EMT proteins (E-cadherin and Vimentin) expression was measured using Western blot analysis, the detection results implied that there was no significant difference in E-cadherin and Vimentin expression between the blank group and the mimics NC group (*P *> 0.05); relative to the blank group, Vimentin expression in the miR-205 mimics group was elevated, while the expression of E-cadherin was reduced (*P *< 0.05, Fig. 7a). No evident difference could be found in the expression of E-cadherin and Vimentin between the exo-blank group and the exo-mimics NC group (*P *> 0.05); in contrast to the exo-blank group, the expression of Vimentin in the exo-miR-205 mimics group was enhanced, while the expression of E-cadherin was declined (*P *< 0.05, Fig. 7b).

The expression of AKT, p-AKT, mTOR and p-mTOR was evaluated by Western blot analysis, the outcomes suggested that after SKOV3 cells were directly transfected with miR-205 mimics, no marked difference could be found in the expression of p-AKT/AKT and p-mTOR/mTOR in SKOV3 cells between the blank group and the mimics NC group (*P *> 0.05); relative to the blank group, the expression of p-AKT/AKT and p-mTOR/mTOR in SKOV3 cells was heightened in the miR-205 mimics group (*P *< 0.05, Fig. 7c).

Western blot analysis was performed to evaluate the expression of p-AKT/AKT and p-mTOR/mTOR after 24-h co-culture, the results indicated that there was no noticeable difference in expression of p-AKT/AKT and p-mTOR/mTOR in SKOV3 cells between the exo-blank group and the exo-mimics NC group (*P *> 0.05); contrasted to the exo-blank group, expression of p-AKT/AKT and p-mTOR/mTOR in SKOV3 cells was enhanced in the exo-miR-205 mimics group (*P *< 0.05, Fig. 7d).

### VEGFA is a target gene of miR-205 and was down-regulated via overexpressed miR-205

**Fig. 8:**
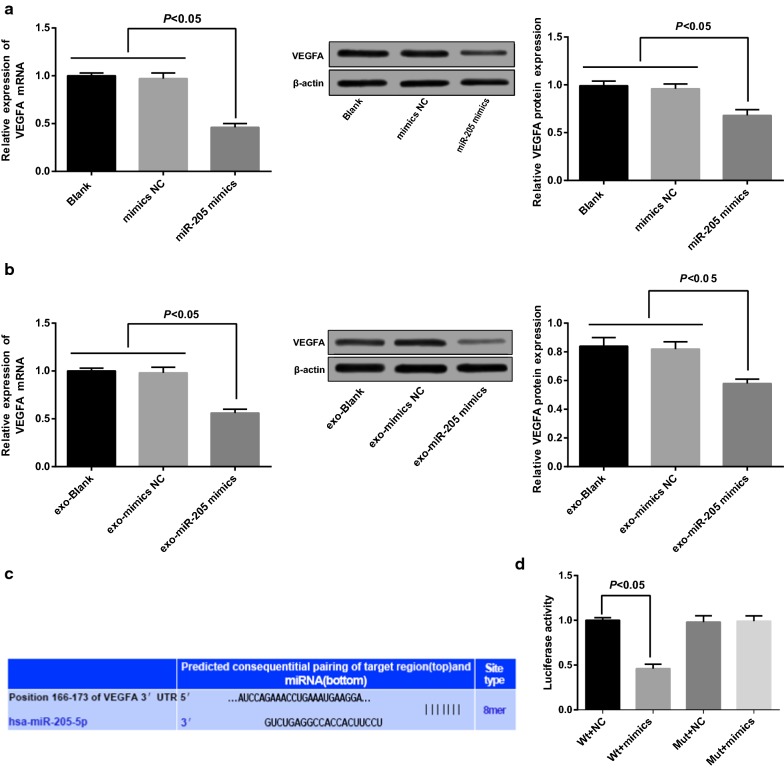
VEGFA is a target gene of miR-205 and was down-regulated via overexpression of miR-205. **a** Detection of VEGFA expression in each group after SKOV3 cells was transfected with miR-205 mimic; **b** detection of VEGFA expression of receptor SKOV3 cells in each group; **c** the target site of VEGFA and relative miR-205 was confirmed by Target Scan; **d** results of dual-luciferase reporter gene assay

The expression of mRNA and protein of VEGFA was evaluated by RT-qPCR and Western blot analysis. The outcomes clarified that there was no observable difference in mRNA and protein expression of VEGFA between the blank group and the mimics NC group (*P *> 0.05); in comparison to the blank group, mRNA and protein expression of VEGFA was declined in the miR-205 mimics group (*P *< 0.05, Fig. 8a). No apparent difference could be observed in the expression of mRNA and protein of VEGFA between the exo-blank group and the exo-mimics NC group (*P *> 0.05); contrasted to the exo-blank group, mRNA and protein expression of VEGFA in the exo-miR-205 mimics was declined (*P *< 0.05, Fig. 8b).

The target site of VEGFA and relative miR-205 was confirmed by an online prediction software Target Scan, and sequence was shown in Fig. 8c. To prove that it was the predicted binding site of miR-205 that resulted in a change of luciferase activity, the reporter plasmids were respectively inserted into the Wt sequence and the Mut sequence, of which VEGFA 3′UTR was lack of the binding site of miR-205. Dual-luciferase reporter gene assay was conducted, the SKOV3 cells were co-transfected with miR-205 mimics and Wt-miR-205/VEGFA or Mut-miR-205/VEGFA, the outcomes revealed that miR-205 mimics exerted an inapparent impact on the luciferase activity of Mut-miR-205/VEGFA, the luciferase activity of Wt-miR-205/VEGFA was noticeably attenuated (*P *< 0.05, Fig. 8d).

## Discussion

The recurrence of ovarian cancer was a main scientific and clinical barrier to cancer control. Few symptoms could perform in the early stages of ovarian cancer, and most of the ovarian cancer patients were diagnosed with ovarian cancer in advanced stages [7]. What has been verified is that the miRNAs, that were characterized as small non-coding RNAs, played a part of leading molecules in the RNA silencing [21]. Moreover, a study has unraveled that miR-205 performed an ectopic expression in another gynecologic malignancy, endometrial cancer [22]. Interestingly, the up-regulation of miR-205 has also been identified in ovarian cancer according to a recent study [13]. Nevertheless, there is little known about miR-205 and exosomes in the progression of ovarian cancer. Consequently, this study was determined to assess the impacts of miR-205 and exosomes on ovarian cancer cells, and we have found that miR-205 may act as a proto-oncogene in ovarian cancer development. Exosomes from donor SKOV3 cells shuttled miR-205 could participate in the proliferation, migration, invasion, apoptosis as well as EMT progression of receptor SKOV3 cells through targeting VEGFA.

One of the most vital findings in our study suggested that miR-205 was up-regulated in ovarian cancer. Except for the diseases mentioned earlier, the high expression of miR-205 has also been proved in other human diseases, such as inflammatory breast cancer [23] and endometrial carcinoma [24]. Furthermore, it was obvious in our research that overexpressed miR-205 could promote proliferation and attenuate apoptosis of ovarian cancer cells. In consistent with this result, Hong Xie et al. have revealed that overexpressed miR-205 in human cervical cancer cells has the ability of increasing the cell proliferation [25]. Another study has provided evidence to prove that overexpressed miR-205 could promote the proliferation and decrease apoptosis of nasopharyngeal carcinoma cells [26], which is in line with the outcomes of this research. A recent study has revealed that miR-205 participated in in EMT process [16]. Jin et al. have unveiled that the overexpression of miR-205 inhibited E-cadherin expression and promoted Snail expression in endometrial carcinoma cells [27]. Moreover, Wang et al. have discovered that miR-205 inhibitor could increase the expression of E-cadherin and decrease the expression of Snail2 and Vimentin [28], and it has been unraveled that miR-205 overexpression could down-regulate E-cadherin and up-regulated Snail expression [26]. It has been uncovered by Duan et al. that the inhibited miR-205 could restrain the expression of MMP-2 and MMP-9 [29]. Results of our research unearthed that after miR-205 was overexpressed, the levels of MMP-2, MMP-9, and Vimentin were elevated, and the expression of E-cadherin was reduced, which were in line with the published researches.

Another significant outcome of our study is that overexpressed miR-205 could down-modulate VEGFA expression. Similarly, the target relation between miR-205 and VEGFA has also been unraveled in patients with breast cancer [30]. What’s more, exosomes from donor ovarian cancer cell SKOV3 shuttled miR-205 could advance the migration as wells as invasion of ovarian cancer cells via targeting VEGFA. The similar results in a previous research proved that miR-205 was implicated in the progression of ovarian cancer via targeting VEGFA, and was able to inhibit the invasion of ovarian cancer cells [14]. The correlation between exosomal overexpressed miR-205 and EMT has been uncovered in our research that overexpression of miR-205 has the capacity to modulate the progression of EMT in ovarian cancer. Chang Xu et al. have given out confirmation that miR-205 could suppress EMT of gastric cancer cells [31]. It has been proved that some of the patient-derived ovarian cancer effusion exosomes might have clinical relevance [32]. Additionally, a study has revealed that the let-7 family, which is known to restrict cell proliferation, was overexpressed in exosomes from SKOV3 cells. Meanwhile, the miR-200 family, which is able to repress EMT, was only expressed in exosomes derived from OVCAR3 cells [6]. The promotive role of VEGFA in EMT process has also been unveiled in a recent study [33], which suggested that VEGFA could elevate the tumor-initiating stem cell population in different cancers, and also induce EMT and metastasis. All of these data have further confirmed the mechanism and function of miR-205, VEGFA and exosomes in human diseases.

## Conclusion

In conclusion, our study provides evidence that miR-205 may act as a proto-oncogene in the progression of ovarian cancer. Exosomes from donor ovarian cancer cell SKOV3 shuttled miR-205 could affect the proliferation, migration, invasion, apoptosis and EMT progression of receptor SKOV3 cells via regulating VEGFA, making miR-205 to be a potential diagnostic biomarker of ovarian cancer. However, the specific interaction between miR-205 and VEGFA has not been completely unraveled in our study. Thus, more efforts are remained to further assess the function mechanisms of miR-205, its target gene VEGFA, and exosomes in the progression of ovarian cancer.

## Data Availability

Not applicable.

## Abbreviations

miRNAs: microRNAs
EMT: epithelial–mesenchymal transition
FIGO: Federation International of Gynecology and Obstetrics
PBS: phosphate buffered solution
EdU: 5-ethynyl-2′-deoxyuridine
PI: propidium iodide
EDTA: ethylene diamine tetraacetic acid
PVDF: polyvinylidene fluoride
TBST: tris buffer solution with tween
ANOVA: analysis of variance
LSD: least significant difference method
OR: odds ratio
ROC: receiver operating characteristic
AUC: area under curve

## References

[1] Gao MQ (2010). CD24+ cells from hierarchically organized ovarian cancer are enriched in cancer stem cells. Oncogene.

[2] van Dam GM (2011). Intraoperative tumor-specific fluorescence imaging in ovarian cancer by folate receptor-alpha targeting: first in-human results. Nat Med.

[3] Kazerouni N (2006). Family history of breast cancer as a risk factor for ovarian cancer in a prospective study. Cancer.

[4] Bull CJ, Yarmolinsky J, Wade KH (2016). Commentary: mendelian randomization analysis identifies circulating vitamin D as a causal risk factor for ovarian cancer. Int J Epidemiol.

[5] Delort L (2009). Central adiposity as a major risk factor of ovarian cancer. Anticancer Res.

[6] Kobayashi M (2014). Ovarian cancer cell invasiveness is associated with discordant exosomal sequestration of Let-7 miRNA and miR-200. J Transl Med.

[7] Bhattacharya R (2009). MiR-15a and MiR-16 control Bmi-1 expression in ovarian cancer. Cancer Res.

[8] Liu G (2014). MiR-506 suppresses proliferation and induces senescence by directly targeting the CDK4/6-FOXM1 axis in ovarian cancer. J Pathol.

[9] Wiklund ED (2011). Coordinated epigenetic repression of the miR-200 family and miR-205 in invasive bladder cancer. Int J Cancer.

[10] Mondal G (2017). EGFR-targeted cationic polymeric mixed micelles for codelivery of gemcitabine and miR-205 for treating advanced pancreatic cancer. Mol Pharm.

[11] Childs G (2009). Low-level expression of microRNAs let-7d and miR-205 are prognostic markers of head and neck squamous cell carcinoma. Am J Pathol.

[12] Dusilkova N (2017). Plasma miR-155, miR-203, and miR-205 are biomarkers for monitoring of primary cutaneous T-cell lymphomas. Int J Mol Sci.

[13] Li J (2017). Upregulation of MiR-205 transcriptionally suppresses SMAD4 and PTEN and contributes to human ovarian cancer progression. Sci Rep.

[14] Wei J (2017). MicroRNA-205 promotes cell invasion by repressing TCF21 in human ovarian cancer. J Ovarian Res.

[15] Wang W (2019). The value of plasma-based MicroRNAs as diagnostic biomarkers for ovarian cancer. Am J Med Sci..

[16] Zhang M (2019). Sequence diverse miRNAs converge to induce mesenchymal-to-epithelial transition in ovarian cancer cells through direct and indirect regulatory controls. Cancer Lett.

[17] Li J (2015). The role of miR-205 in the VEGF-mediated promotion of human ovarian cancer cell invasion. Gynecol Oncol.

[18] Dinkins MB (2014). Exosome reduction in vivo is associated with lower amyloid plaque load in the 5XFAD mouse model of Alzheimer’s disease. Neurobiol Aging.

[19] Zhao Z (2016). A microfluidic ExoSearch chip for multiplexed exosome detection towards blood-based ovarian cancer diagnosis. Lab Chip.

[20] Morelli AE (2004). Endocytosis, intracellular sorting, and processing of exosomes by dendritic cells. Blood.

[21] Ha M, Kim VN (2014). Regulation of microRNA biogenesis. Nat Rev Mol Cell Biol.

[22] Karaayvaz M (2012). Prognostic significance of miR-205 in endometrial cancer. PLoS ONE.

[23] Huo L (2016). MicroRNA expression profiling identifies decreased expression of miR-205 in inflammatory breast cancer. Mod Pathol.

[24] Su N (2013). miR-205 promotes tumor proliferation and invasion through targeting ESRRG in endometrial carcinoma. Oncol Rep.

[25] Xie H (2012). miR-205 expression promotes cell proliferation and migration of human cervical cancer cells. PLoS ONE.

[26] Mao Y (2016). MiR-205 promotes proliferation, migration and invasion of nasopharyngeal carcinoma cells by activation of AKT signalling. J Int Med Res.

[27] Jin C, Liang R (2015). miR-205 promotes epithelial–mesenchymal transition by targeting AKT signaling in endometrial cancer cells. J Obstet Gynaecol Res.

[28] Wang B (2016). miR-375 and miR-205 regulate the invasion and migration of laryngeal squamous cell carcinoma synergistically via AKT-mediated EMT. Biomed Res Int.

[29] Duan B (2017). miR-205 as a biological marker in non-small cell lung cancer. Biomed Pharmacother.

[30] Hu Y (2016). miRNA-205 targets VEGFA and FGF2 and regulates resistance to chemotherapeutics in breast cancer. Cell Death Dis.

[31] Xu C (2016). MicroRNA-205 suppresses the invasion and epithelial–mesenchymal transition of human gastric cancer cells. Mol Med Rep.

[32] Vaksman O (2014). Exosome-derived miRNAs and ovarian carcinoma progression. Carcinogenesis.

[33] Kim M (2017). VEGFA links self-renewal and metastasis by inducing Sox2 to repress miR-452, driving Slug. Oncogene.

